# Advances in the synthesis and characterization of phosphorene for bandgap tailoring – a comprehensive review

**DOI:** 10.1039/d4na00886c

**Published:** 2026-02-10

**Authors:** Md. Helal Hossain, Mohammad Asaduzzaman Chowdhury, Md. Touhiduzzaman

**Affiliations:** a Department of Mechanical Engineering, Dhaka University of Engineering and Technology (DUET) Gazipur Dhaka 1707 Bangladesh helalhossain9719@gmail.com; b Department of Industrial and Production Engineering, Dhaka University of Engineering and Technology (DUET) Gazipur 1707 Bangladesh

## Abstract

Phosphorene is a two-dimensional (2D) material obtained from black phosphorus and has attracted considerable attention for its outstanding electronic properties, such as tunable bandgap and high carrier mobility. This review presents the structural and morphological study of direct bandgap phosphorene by atomic force microscopy (AFM), scanning electron microscopy (SEM), and transmission electron microscopy (TEM), which verified the successful exfoliation and uniformity of the nanosheets. Also, the crystalline studies and optical properties investigated through Raman spectroscopy, X-ray diffraction (XRD) and ultraviolet-visible (UV-Vis) spectroscopy have been presented. Photoluminescence (PL) spectroscopy can measure the bandgap energy, demonstrating tunability which is dependent on the number of layers. The increase from 0.33 eV in the bulk to 1.88 eV in bilayers showcases phosphorene's band gap evolution in large-scale synthesis, with a higher-energy transition from 2.0 eV to 3.23 eV highlighting its unique optoelectronic properties. The characterization of phosphorene underscores its promising attributes for bandgap formation, with potential applications ranging from transistors to photodetectors.

## Abstract 2

Phosphorene is a two-dimensional (2D) material obtained from black phosphorus and has attracted considerable attention for its outstanding electronic properties, such as tunable bandgap and high carrier mobility.

## Graphical abstract

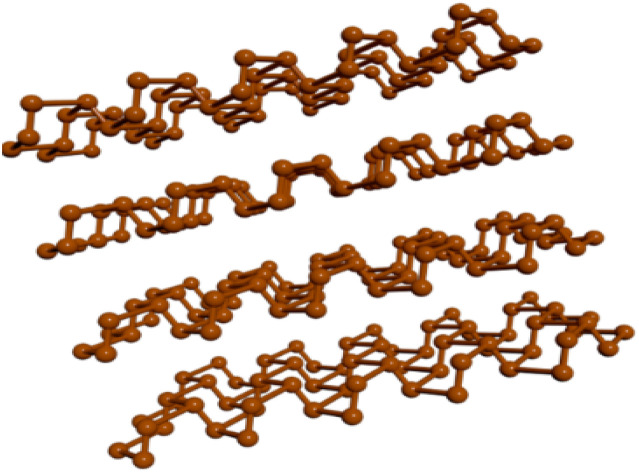

## 1. Introduction

In recent decades, 2D materials have become an intriguing category of substances characterized by unique properties resulting from quantum confinement effects and a significantly higher surface-to-volume ratio.^1^ The current 2D family comprises around one hundred members, this group includes notable materials such as graphene,^2,3^ hexagonal boron nitride (h-BN),^4^ transition metal dichalcogenides (TMDs),^5,6^ silicone,^7,8^ germanene,^9^ stanine,^10^ and others.^11,12^ Phosphorene, an atomically thin form of phosphorus, stands out among all 2D materials due to its exceptional combination of remarkable properties. An essential benefit of phosphorene is its adjustable direct band gap. The band gap can be optimized by manipulating variables like thickness, strain, functionalization, and defect engineering.^13,14^ The black phosphorus (BP) bandgap is layer-dependent, ranging from 0.3 to 1.5 eV, and decreases as the material shifts from the bulk to monolayer. It spans the range of wavelengths from the mid-infrared to visible light, bridging the difference in energy levels between graphene (which has no energy gap) and TMDs (which have an energy gap in the visible light range). No other 2D materials exhibit such a diverse spectrum of bandgap variations. Another benefit is its comparatively high charge carrier mobility, which surpasses 1000 cm^2^ V^−1^ s^−1^ at typical temperatures. The mobility value of this is significantly greater than that of TMDs. Furthermore, phosphorene exhibits inherent ambipolar behavior, unlike TMDs, which only exhibit n-type semiconductor behavior. Phosphorene has the ability to operate as either a p-type or n-type semiconductor. These characteristics make phosphorene one of the most promising candidates in various fields, such as high-performance digital applications,^15^ ultrafast laser,^16^ bio-photonics,^17^ energy storage devices,^18^ optoelectronics,^19^ solar-cells,^20^ and nano-sensors.^21^

The achievements of black phosphorene have catalyzed the development of two-dimensional layered phosphorus allotropes. Significant advancements in first-principles theoretical methodologies have proposed numerous unique phosphorus allotropes with diverse electrical characteristics. The practical production of 2D phosphorus is progressing slower than the advancement of theoretical calculations. Thus far, apart from α-P (black phosphorene) and β-P (blue phosphorene),^22,23^ phosphorene pentamers,^24^ violet phosphorene,^25^ and ultrathin red phosphorus^26^ nanosheets have been successfully created. However, experimental studies on the synthesis of additional phosphorene variants remain limited.

Various effective techniques have been devised for the production of phosphorene, including liquid-phase exfoliation,^27,28^ electrochemical exfoliation,^29,30^ chemical vapor deposition (CVD)/chemical vapor transport (CVT),^31,32^ pulsed laser deposition,^33^ thermal thinning,^34^ and others.

Phosphorene, a well-studied material, has been employed in various applications, including transistors^35,36^ and optoelectronics,^37,38^ gas sensors,^39,40^ chemo-sensors,,^41^ biosensors,^42,43^ energy storage,^44^ and photocatalysts.^45^ Phosphorene exhibits highly favorable electrochemical characteristics, making it well-suited for various electrochemical applications.^46^ Nevertheless, two significant inherent constraints hinder the widespread use of phosphorene in various practical applications.^47^ One challenge with phosphorene is its limited long-term stability under standard environmental settings. However, recent advancements in capping technology, influenced by the microelectronics sector, are working towards resolving this problem.^48^

This review examines the methods and techniques for synthesizing single-layer and few-layer phosphorene, with particular emphasis on the current advancements and challenges associated with the epitaxial growth of phosphorene. It delves into synthesis strategies, physical properties, and applications of phosphorene and its derivatives, considering the suitability of the atomic structure and the properties of phosphorus. The review addresses the existing and emerging challenges in phosphorene synthesis, mainly focusing on improving crystal quality and achieving large-size crystals to enhance electronic, transport, and optical properties. Finally, it provides an outlook on future research directions to investigate phosphorene synthesis strategies further and enhance phosphorene performance. The review seeks to offer insights into the progress in developing high-quality phosphorene and its applications in various scientific and technological domains.

## 2. Phosphorene

**Fig. 1:**
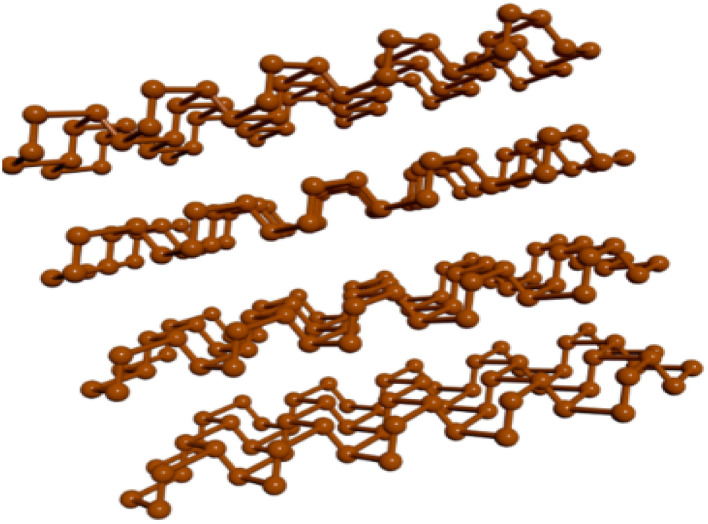
Three-dimensional structure of bulk orthorhombic black phosphorus in the AA stacking configuration. A single layer is referred to as phosphorene (2D phosphane). Reproduced from ref. 49 with permission from Nickclark332 under Creative Commons Attribution–ShareAlike 4.0 License.

Phosphorene, from black phosphorus, features a unique puckered structure that endows it with exceptional electronic and mechanical properties. Fig. 1 illustrates the basic atomic structure of phosphorene.^49^ It features a direct bandgap, ideal for optoelectronic applications such as transistors and photodetectors. Known for high carrier mobility and flexibility, phosphorene is promising for flexible electronics and advanced sensors. However, its instability in the air due to oxidation poses a significant challenge, requiring protective encapsulation for practical use. Research focuses on enhancing its stability and scalability and exploring new energy storage and photovoltaics applications.

## 3. Synthesis methods of phosphorene

**Fig. 2:**
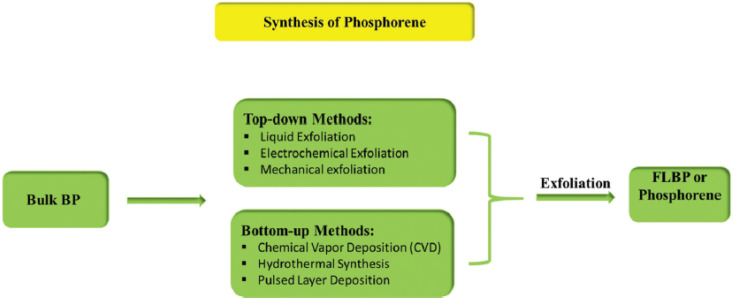
Different preparative methods of few-layer black phosphorus (FLBP) or phosphorene, reproduced from ref. 50 with permission from the Royal Society of Chemistry, *Materials Advances*, 2022, 3, 1234–1248 (DOI: 10.1039/d1ma01101d), © 2022 RSC.

Various methods are employed to synthesize phosphorene, with its quality and potential applications mainly depending on the fabrication processes. Fig. 2 presents a flowchart detailing some of these synthesis methods.

### 3.1 Solution based intercalation

**Fig. 3:**
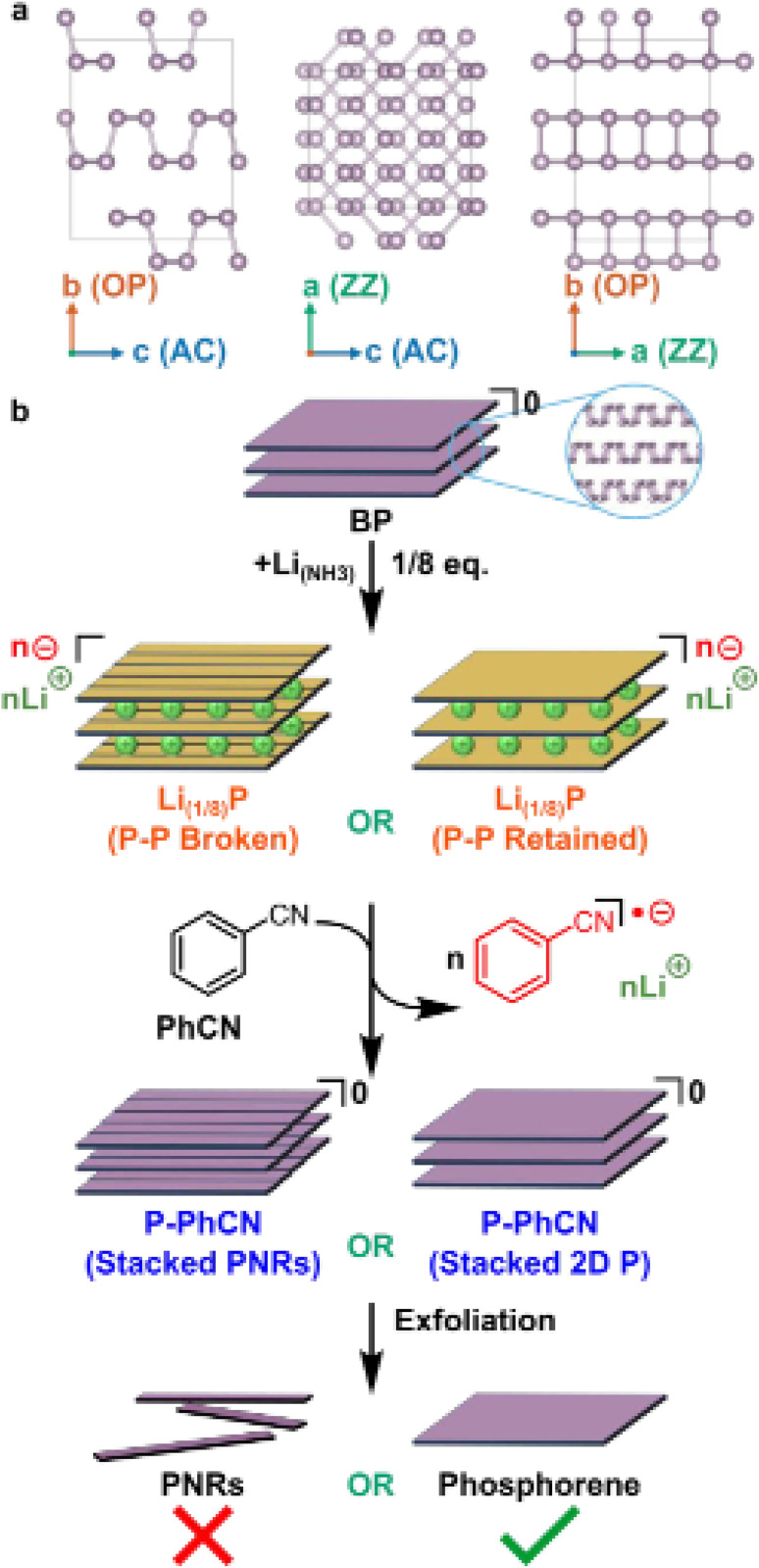
(a) Black phosphorus (BP) depicted along its crystallographic axes. (b) Schematic illustration of the lithium intercalation process of black phosphorus forming Li_1/8_P, resulting in the formation of phosphorene nanoribbons (PNRs) during the intercalation process. Adapted from ref. 53 with permission from the Royal Society of Chemistry, *Nanoscale*, 2024, 16, 1742–1750, copyright 2024. This Open Access Article is licensed under a Creative Commons Attribution 3.0 Unported Licence.^53^

Fig. 3 shows the widely used technique for producing phosphorene nanoribbons (PNRs) at the lab scale, which involves peeling black phosphorus (BP) with adhesive tape and applying electron beam lithography. This approach produces PNRs with precise lengths and uniform width along the ribbons. Recent advancements have demonstrated that tape exfoliation can directly produce phosphorene nanoribbons (PNRs) by partially separating the surface layer of few-layer black phosphorus (BP), achieving widths as small as 14 nm. These ribbons are then collected using polydimethylsiloxane.^51^ Nevertheless, scaling up the combination of tape exfoliation and electron beam lithography is challenging due to the intricate process required to synthesize each individual phosphorene nanoribbon (PNR). Fig. 3 illustrates the crystallographic structure of black phosphorus (BP) and its intercalation process with lithium, resulting in the formation of phosphorene nanoribbons (PNRs).

An alternative method that offers scalability involves chemical vapor transport. This method produces narrow, high aspect ratio black phosphorus (BP) columns derived from BP crystals. These columns are then stripped away using sonication in an amide-based solvent. Although this method shows promise for scalable production of PNRs, the thinnest structures obtained consist of multiple layers and have widths of approximately 1 µm, commonly referred to as ‘nanobelts’.^52^ These dimensions are inadequate for effective lateral confinement of phosphorene nanoribbons (PNRs), which are anticipated to result in properties akin to those of few-layer 2D phosphorene.

### 3.2 Quantum dot method

**Fig. 4:**
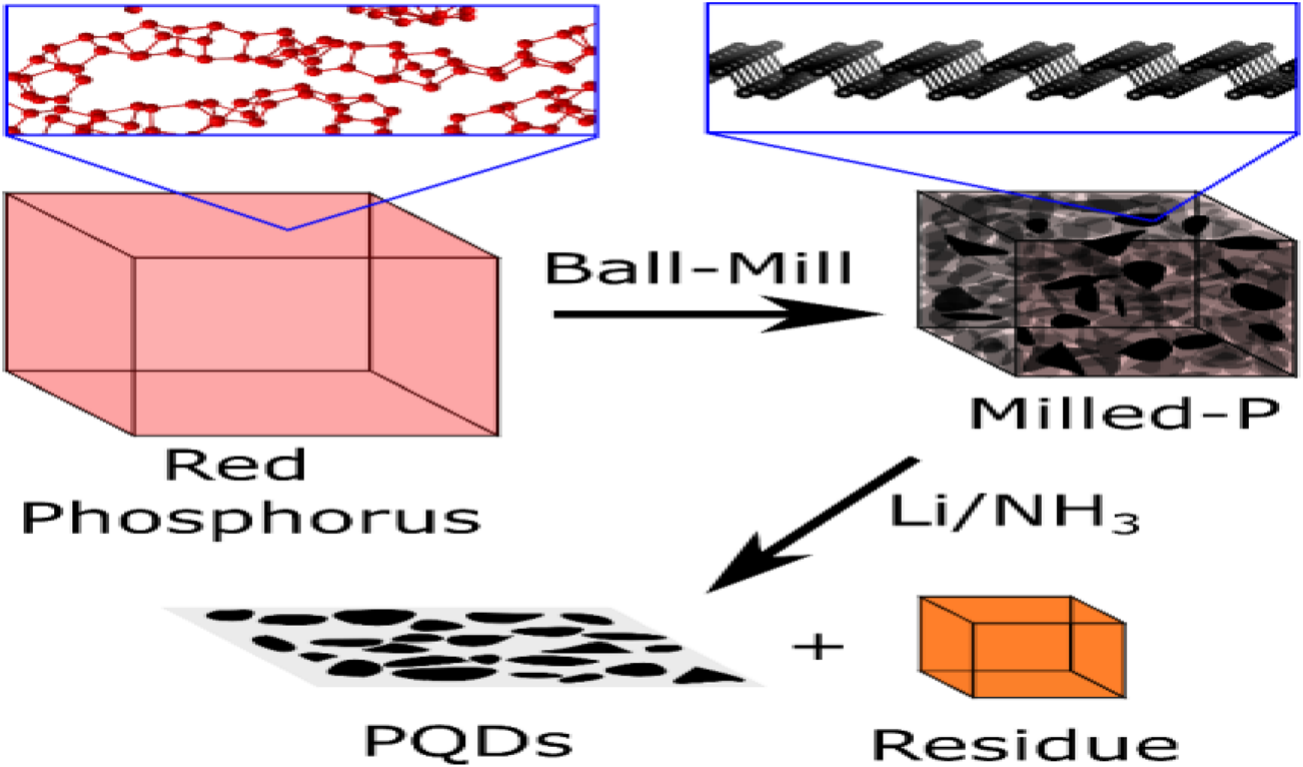
Diagram illustrating the conversion of amorphous red phosphorus (Pred_\text{red}red) into phosphorus quantum dots (PQDs) *via* ball-milling followed by reductive etching in a lithium electrode. Adapted from ref. 57 with permission from Wiley-VCH GmbH, *Chemistry – A European Journal*, 2023, 29, e202301232, © 2023 The Authors. This Open Access Article is licensed under the terms of the Creative Commons Attribution License.^57^

Black phosphorus (Pblack) is a phosphorus allotrope; it is a van der Waals layered material made up of corrugated layers where each phosphorus atom is bonded to three neighboring atoms *via* two short P–P bonds and one longer P–P bond.^54^ Nanomaterials derived from the 3D parent structure of black phosphorus (Pblack), such as phosphorene (2D), phosphorene nanoribbons (PNRs, 1D), and phosphorene quantum dots (PQDs, 0D), have demonstrated significant potential for use in energy harvesting and storage applications.^55^ Recent efforts to scale up the production of black phosphorus (Pblack) using ceramic processing techniques have produced high-density nanocrystalline ‘ceramics’ with black phosphorus (Pblack) domains under 50 nm at a 10 gram scale. These materials can be further reduced into smaller clusters using solvent-assisted shear force exfoliation. Again, red phosphorus (Pred) can be converted into Pblack by ball-milling under an inert atmosphere; by applying high local pressure and temperature, the phase transition to more stable, higher-density black phosphorus (Pblack) crystallites can be facilitated. However, ball-milling single-crystal black phosphorus (Pblack) can lead to degradation because of localized bond shearing; this results in the formation of amorphous Pred regions, indicating that this method may not produce highly crystalline microscale Pblack/phosphorene. Fig. 4 shows amorphous red phosphorus (Pred) conversion into phosphorus quantum dots (PQDs) through ball-milling and reductive etching in a lithium electrode. Ball-milling in a liquid medium can directly generate small phosphorus quantum dots (PQDs) (∼5 nm) from Pred, with at least 50% of the phosphorus atoms bonded to oxygen.^56^ For scalable and cost-effective production of PQDs with reduced oxygen content, a two-step process is used: first, dry ball-milling of Pred to produce nanocrystalline black phosphorus (Pblack), then isolation of PQDs through reductive etching.

### 3.3 Chemical vapor transport

**Fig. 5:**
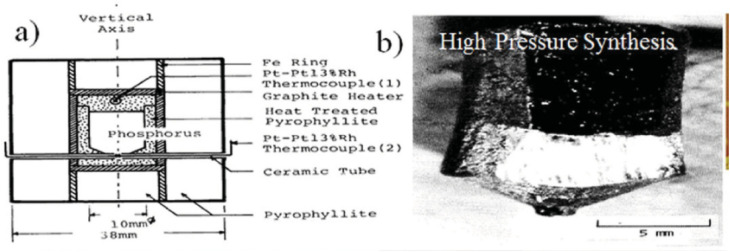
(a) Growth of black phosphorus (BP) crystals under high pressure and high temperature conditions. (b) Optical image of a BP crystal showing distinct and well-defined crystal faces. Adapted from ref. 60, *Nat. Nanotechnol.*, 2014, 9, 372–377. This figure is reused in an Open Access publication with appropriate acknowledgment.^60^

Fig. 5 illustrates the growth of black phosphorus (BP) crystals at high pressure and temperature, along with the distinct, clear faces of the BP crystals. Black phosphorus (BP) single crystals can be synthesized using various methods, including high-pressure synthesis; black phosphorus (BP) single crystals can be synthesized using techniques such as chemical vapor transport (CVT), mercury catalysis, and liquid bismuth recrystallization. White phosphorus (WP) can be transformed into BP single crystals by applying a hydrostatic pressure of 1.2 GPa at 200 °C for 5–30 minutes.^58^ This transformation was discovered serendipitously while attempting to convert white phosphorus (WP) into red phosphorus (RP) using high pressure and moderate temperatures. This process was subsequently refined to produce large BP crystals (Fig. 5a and b). In high-pressure synthesis, red phosphorus (RP) is initially heated to 1000 °C, then slowly cooled to 600 °C at a rate of 100 °C per hour while maintaining a constant pressure of 10 kbar. This technique produces BP crystals of high quality and purity. Endo *et al.* successfully produced a single layer larger than 5 × 5 × 10 mm^3^, which was grown by melting the material at 900 °C under a hydrostatic pressure of 1 GPa.^59^

Other techniques, including mercury catalysis and liquid bismuth recrystallization, are also employed to produce BP crystals. These methods generally yield needle or plate-shaped black phosphorus (BP) single crystals with sizes around 5 mm in length and thicknesses ranging from 10 to 100 µm. However, these techniques are limited due to their toxicity and the considerable time required for the synthesis process.

### 3.4 Liquid exfoliation

**Fig. 6:**
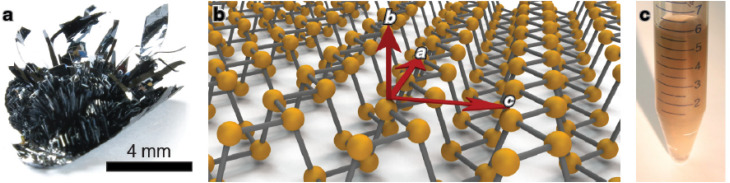
(a) Black phosphorus. (b) Schematic illustration of phosphorene monolayer orientations along the armchair (‘a’), zigzag (‘b’), and normal (‘c’) directions. (c) Suspension of two-dimensional phosphorus in isopropanol after liquid exfoliation. Reproduced from ref. 66 with permission from the American Physical Society, *Physical Review Materials*, 2022, 6, 044002, © 2022 American Physical Society.^66^

Fig. 6 presents black phosphorus, a phosphorene monolayer's orientations, and a suspension of 2D phosphorus in isopropanol following liquid exfoliation. Black phosphorus can undergo liquid exfoliation to produce various two-dimensional flakes with tunable optical properties similar to quantum dots. Black phosphorus, a crystalline variant of phosphorus with layers arranged in a three-dimensional pattern (Fig. 6a),^61^ and its two-dimensional variant, phosphorene (Fig. 6b),^62^ have recently regained interest. Phosphorene, a two-dimensional material featuring a distinctive corrugated or accordion-like structure, has garnered considerable theoretical attention because of its anticipated anisotropic and thickness-dependent properties. These include its optoelectronic behavior, mechanical strength, and chemical reactivity.^63,64^ Despite a reliable method for producing or purifying monolayer or few-layer phosphorene, most predictions in this field have yet to be confirmed. Monolayers are often located at the edges of thicker sheets and are typically too small for comprehensive characterization. The practical limitations mentioned stem from intrinsic issues related to phosphorus. It is noteworthy that phosphorus–phosphorus bonds are significantly weaker compared to carbon–carbon bonds. Furthermore, research has revealed the material's propensity to oxidize or transition into various allotropes.

Furthermore, the interlayer interactions in black phosphorus may exhibit greater strength than other two-dimensional materials. Strong interlayer contacts hinder the exfoliation process, making it more challenging to separate black phosphorus into thin layers. Consequently, black phosphorus is more prone to fragmentation than other two-dimensional materials.^65^

### 3.5 Electrochemical exfoliation

**Fig. 7:**
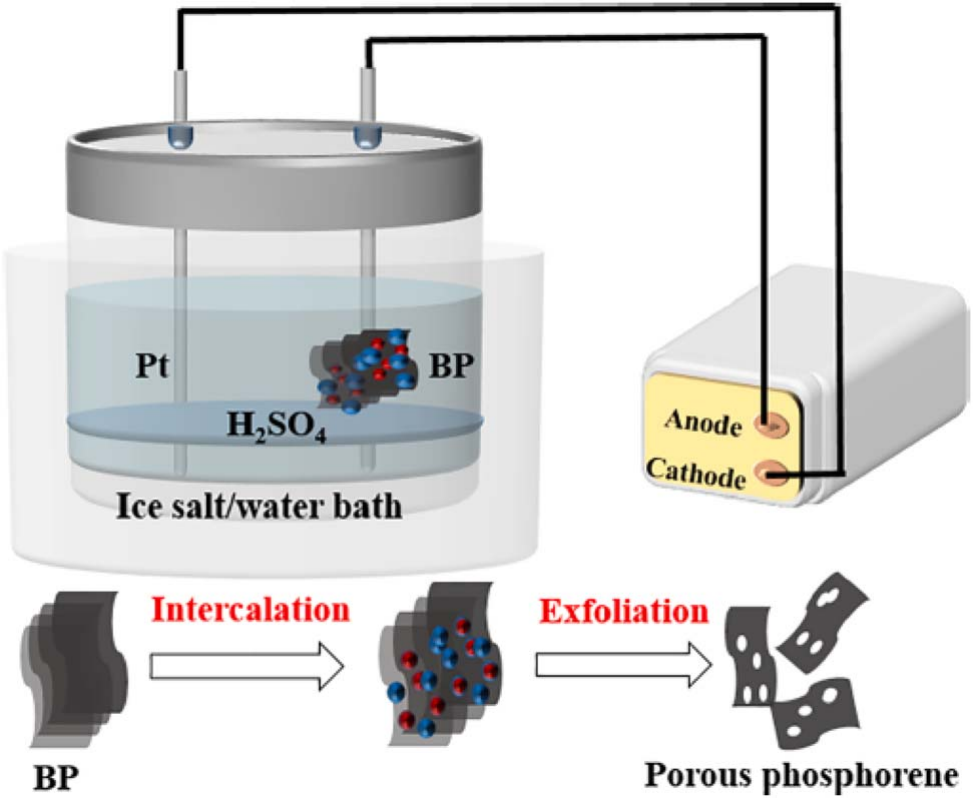
Diagram illustrating the electrochemical exfoliation process for the synthesis of holey phosphorene. Adapted from ref. 68 with permission from the authors, *Electrochemistry Communications*, 2021, 128, 107074. This Open Access Article is licensed under a Creative Commons Attribution-NonCommercial-NoDerivatives 4.0 International (CC BY-NC-ND 4.0) Licence.^68^

As shown in Fig. 7, black phosphorus (BP) was secured to a platinum (Pt) clip, which served as the working anode. Starting at a distance of 1.5 cm, a platinum (Pt) wire is positioned parallel to the black phosphorus (BP), which functions as the cathode. Both electrodes were immersed in an electrolytic cell beaker containing a 1 M H_2_SO_4_ solution. The beaker was placed in an insulated bucket containing either a salt (NaCl) solution or an ice water bath. A DC power supply (MS305D, Dongguan Maihao Electronic Technology Co. Ltd) was used to apply a constant voltage of 3 V or 4 V to the BP electrode. Centrifugation was performed at 3000 rpm for 15 minutes, and the supernatant was then collected from the resulting nano-BP. The supernatant was subjected to a second centrifugation for 15 minutes at 13 000 rpm. Finally, anhydrous ethanol was used to disperse the collected precipitate.^67^

### 3.6 Mechanical exfoliation

**Fig. 8:**
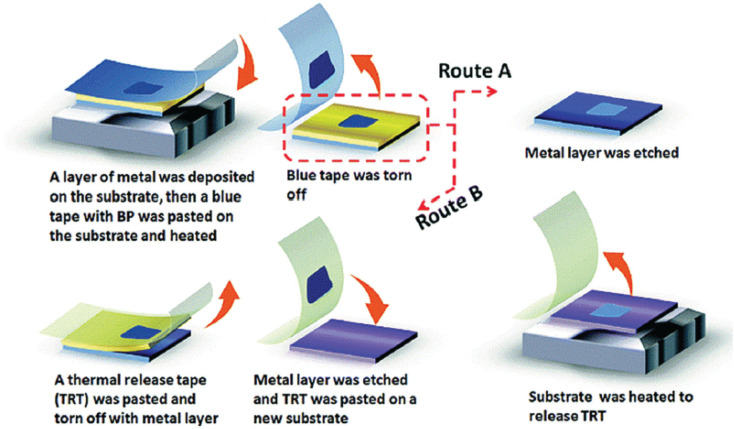
Metal-assisted exfoliation of few-layer black phosphorus (FLBP). Adapted from ref. 74, *Chem. Commun.*, 2018, 54, 595–598.^74^

Fig. 8 illustrates the metal-assisted exfoliation of BP. Mechanical exfoliation is another method for preparing thin, layered materials. Exfoliating black phosphorus (BP) is relatively straightforward because of its weak interlayer van der Waals forces.^69^ This technique, commonly known as Scotch-tape delamination, involves using Scotch Magic tape or blue Nitro tape.^70^ Initially, a monolayer BP nanosheet, approximately 0.85 nm thick, is produced using Scotch tape.^71^ The Si/SiO_2_ substrate is first cleaned with acetone, isopropyl alcohol, and methanol to remove any tape residue before transferring the BP nanosheets onto it. The substrate is heat-treated to remove any remaining solvent, yielding the desired nanosheets. The low efficiency of this method is a notable drawback and the challenge of obtaining large monolayers or few-layered BP (FLBP).^72^

To enhance the process, a layer of Ag or Au is first deposited onto PDMS, Si/SiO_2_, Si wafers, or other substrates using magnetron sputtering. BP is then applied to blue tape and placed onto the coated substrate. After removing the blue tape, the metal coating is heated, and the process must be stripped away to secure the FLBP to the substrate. This method facilitates the transfer of FLBP onto different solvents. This method produces few-layer black phosphorus (FLBP) with a lateral size of up to 450 µm and a significantly larger surface area compared to the conventional Scotch tape exfoliation method. Despite some advantages, this technique encounters issues with unpredictable morphology and limited BP yield.^73^

### 3.7 Sono-chemical synthesis

**Fig. 9:**
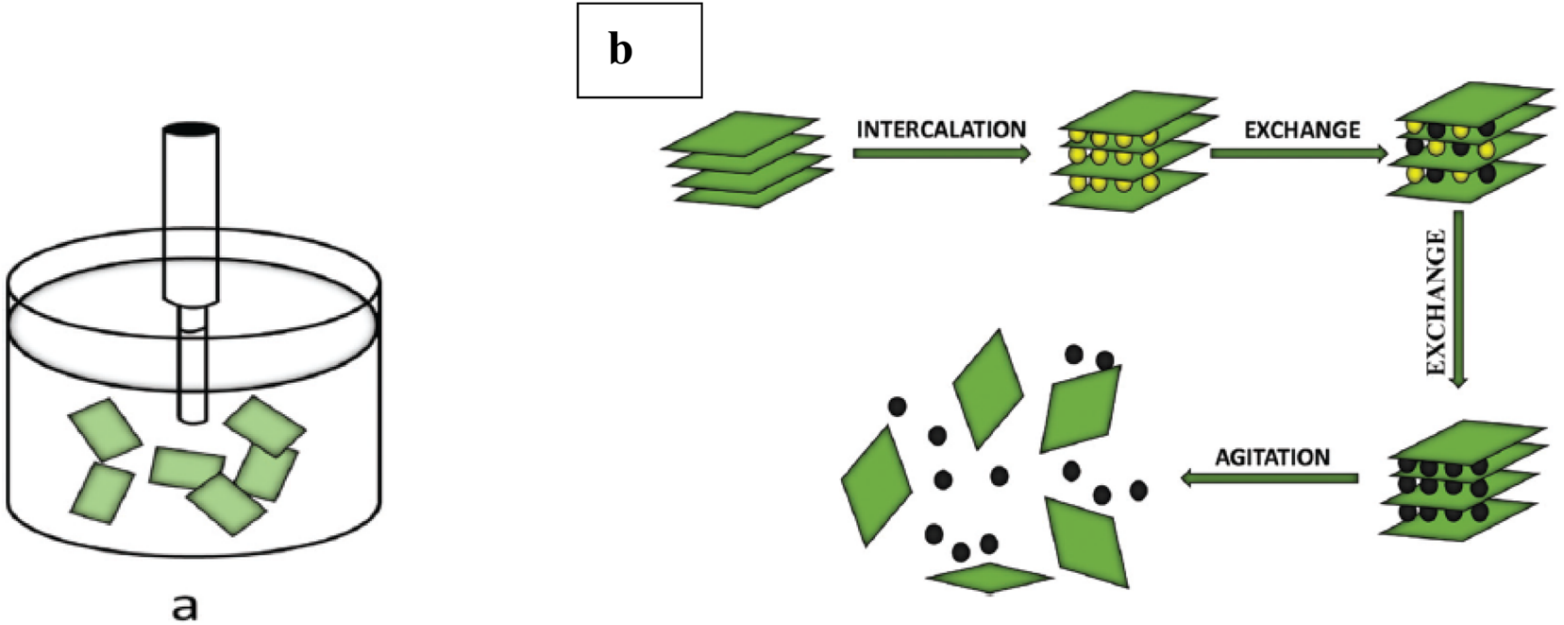
(a) Ultrasonication method, and (b) exfoliation process of black phosphorus (BP) nanosheets. Adapted from ref. 82, *Mater. Adv.*, 2022, 3, 5557–5574. This Open Access Article is licensed under a Creative Commons Attribution-Non Commercial 3.0 Unported Licence.^82^

The ultrasonication method for exfoliating BP involves dispersing BP in an appropriate solvent and then subjecting it to ultrasonic treatment. Centrifugation is used to collect the exfoliated nanosheets. Micro- and nano-sized bubbles during sonication are generated by fluctuations and shear forces. *N*-Methyl pyrrolidone (NMP) and DMSO infiltrate between the BP layers, helping in the distribution of the nanosheets evenly, as depicted in Fig. 9. Choosing an appropriate liquid solvent system for dispersing and exfoliating the material is crucial to carry out liquid-based exfoliation using ultrasonication. Theoretical simulations of surface energy and surface tension offer guidance on effective solvents for exfoliating 2D materials. BP exfoliation efficiency improves as the surface tension of solvents increases. Unlike other two-dimensional materials, producing and separating phosphorene nanosheets effectively requires avoiding water and oxygen, as oxygen atoms tend to chemically bond with phosphorus atoms on the BP nanosheets' surfaces.^75–81^

## 4. Characterization of phosphorene

Characterization of phosphorene involves using advanced techniques to understand its structural, electronic, and optical properties. Raman spectroscopy is commonly employed to assess the vibrational modes of the material and offer valuable insights into layer thickness and crystallinity. Atomic force microscopy (AFM) and scanning electron microscopy (SEM) are employed to study surface topography and determine layer thickness. The chemical composition is revealed by X-ray photoelectron spectroscopy (XPS), along with oxidation states. At the same time, transmission electron microscopy (TEM) offers high-resolution images of internal structures for defect analysis and material integrity.

### 4.1 Raman spectroscopy of phosphorene

**Fig. 10:**
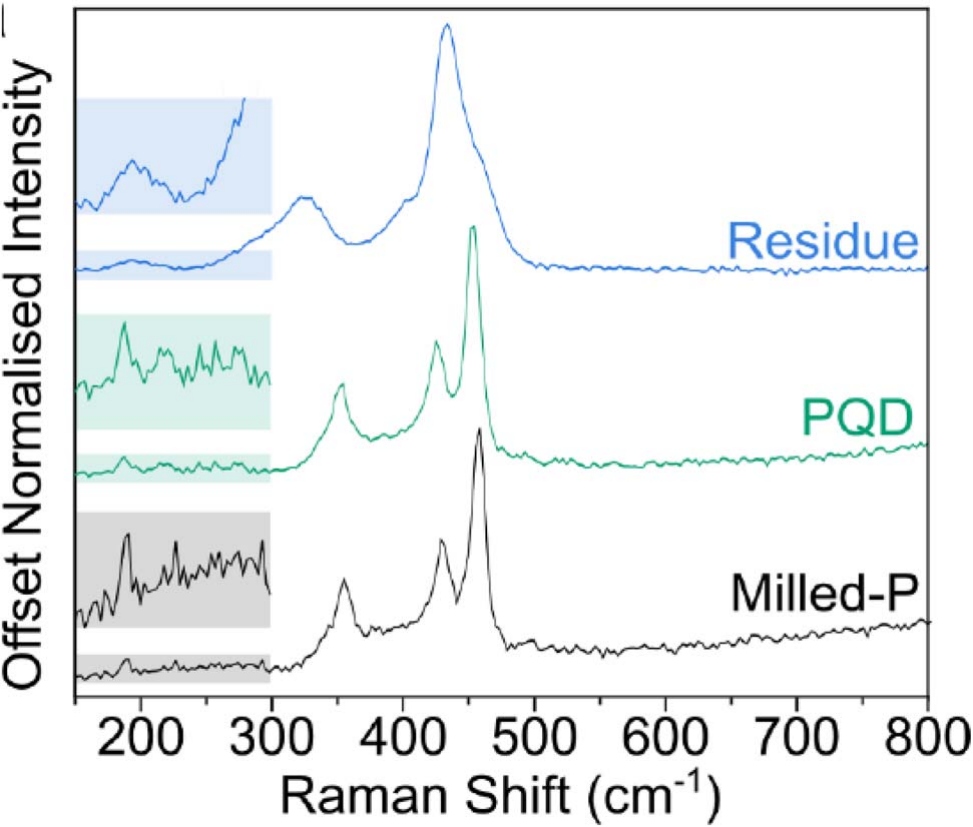
Raman spectroscopy of residue, phosphorus quantum dots (PQDs), and milled-P. Adapted from ref. 83 with permission from the authors, *Chemistry – A European Journal*, 2023, 29, e202301232, © 2023 The Authors. This Open Access Article is licensed under the terms of the Creative Commons Attribution License.^83^

Fig. 10 shows the Raman spectroscopy analysis of residue, phosphorus quantum dots (PQDs), and milled phosphorus (Milled-P). Dry ball-milling of red phosphorus (Pred) was used to obtain black phosphorus powder, known as “Milled-P.” The Ag2, B2g, and Ag1 modes of black phosphorus were consistently observed in the Raman spectra of Milled-P, with characteristic peaks at 457, 428, and 354 cm^−1^, respectively (Fig. 10). This is consistent with other studies on converting Pred to black phosphorus *via* ball-milling. However, the peaks are notably red-shifted by approximately 10 cm^−1^ compared to the highly crystalline black phosphorus. After the ammonia evaporated, a substantial amount of dark orange residue, referred to as “Residue”, around the edges of the flask accumulated as coffee rings. Phosphorus quantum dots (PQDs) were found in the black powder left at the bottom after the process.

### 4.2 X-ray diffraction (XRD) of phosphorene

**Fig. 11:**
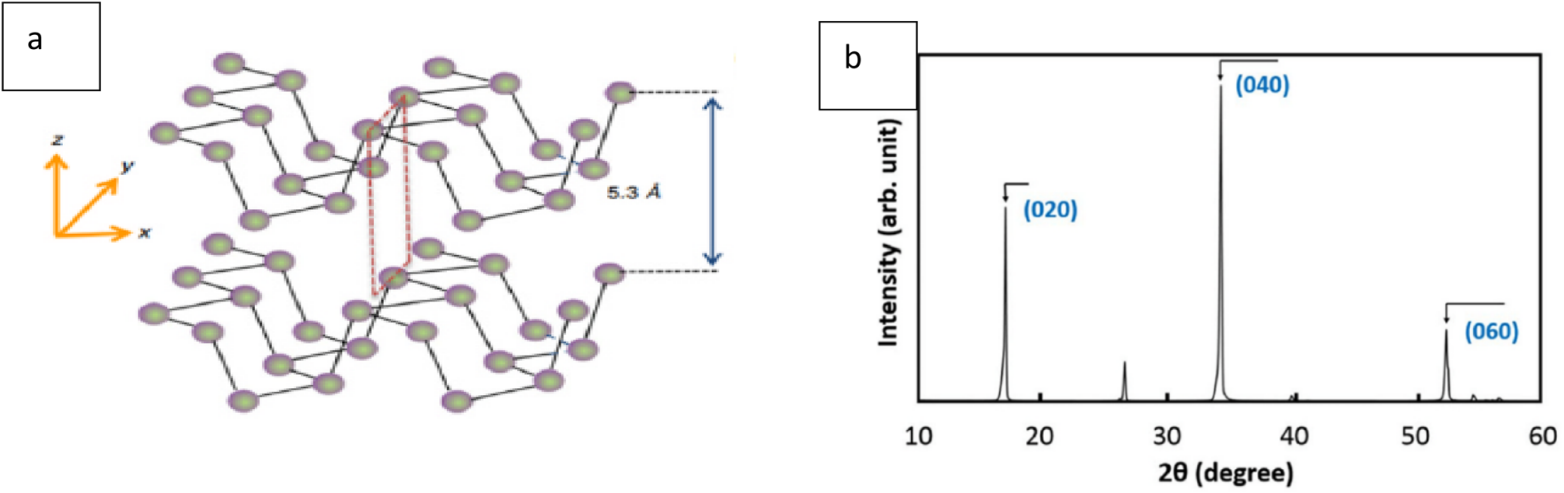
(a) Black phosphorus with its layered crystal structure. (b) XRD pattern of black phosphorus showing peaks corresponding to the (060), (020), and (040) crystal planes. Reproduced from ref. 84 with permission under License ID 1698207-1, *ECS J. Solid State Sci. Technol.*, 2016, 5, © 2016 ECS.^84^

Fig. 11 presents black phosphorus with its layered crystal structure and the XRD pattern displaying peaks for the (060), (020), and (040) crystal planes. Examining the anisotropic properties of black phosphorus (BP) relies on X-ray diffraction (XRD), an essential tool for this purpose (Fig. 11a). XRD plays a critical role in characterizing the structural and anisotropic properties of black phosphorus (BP) and phosphorene. As mentioned, XRD provides key insights into a material's crystal structure. The distinct peaks in the X-ray diffraction (XRD) pattern for black phosphorus (BP) reveal specific structural features (Fig. 11b), enabling researchers to ascertain its lattice parameters and understand the atomic arrangement within the BP crystal structure.

### 4.3 Scanning electron microscopy (SEM) analysis of phosphorene

**Fig. 12:**
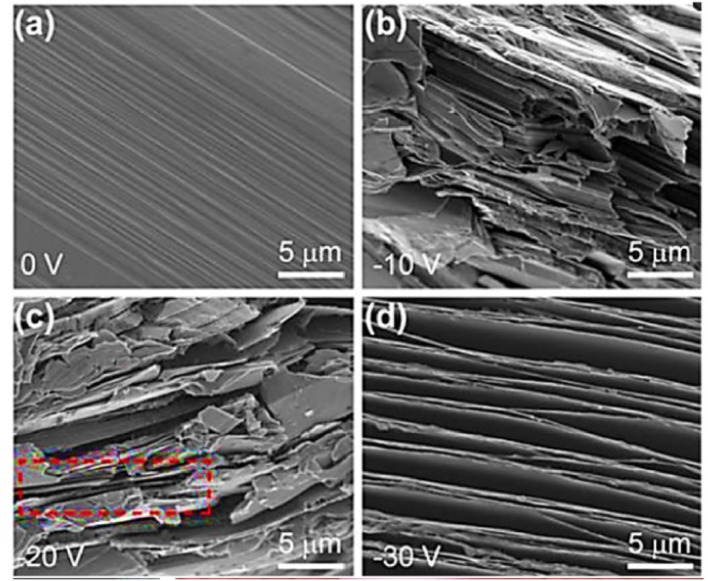
BP crystals exposed to different voltages at 50 °C shown in the SEM images: (a) 0 V, (b) −10 V, (c) −20 V, (d) −30 V. Reproduced from ref. 85 with permission from the American Chemical Society, *ACS Appl. Nano Mater.*, 2019, 2, 6, © 2019 ACS.^85^

Analysis of phosphorene by SEM involves preparing thin, exfoliated samples on conductive substrates. Use an appropriate acceleration voltage (1–30 kV) and adjust the magnification to capture detailed surface morphology, ensuring minimal sample damage and precise imaging. Fig. 12 shows SEM images of BP crystals exposed to different voltages at 50 °C: (a) 0 V, (b) −10 V, (c) −20 V, and (d) −30 V. Coating with a conductive layer may be necessary to reduce charging effects. Two techniques currently attracting interest are electrosynthesis and covalent macromolecular attachment. Fig. 12 depicts these methods. Scanning electron microscopy (SEM) is crucial for examining BP crystals' morphology after exfoliation under different processing conditions (Fig. 12a–d).

### 4.4 X-ray photoelectron spectroscopy (XPS) analysis of phosphorene

**Fig. 13:**
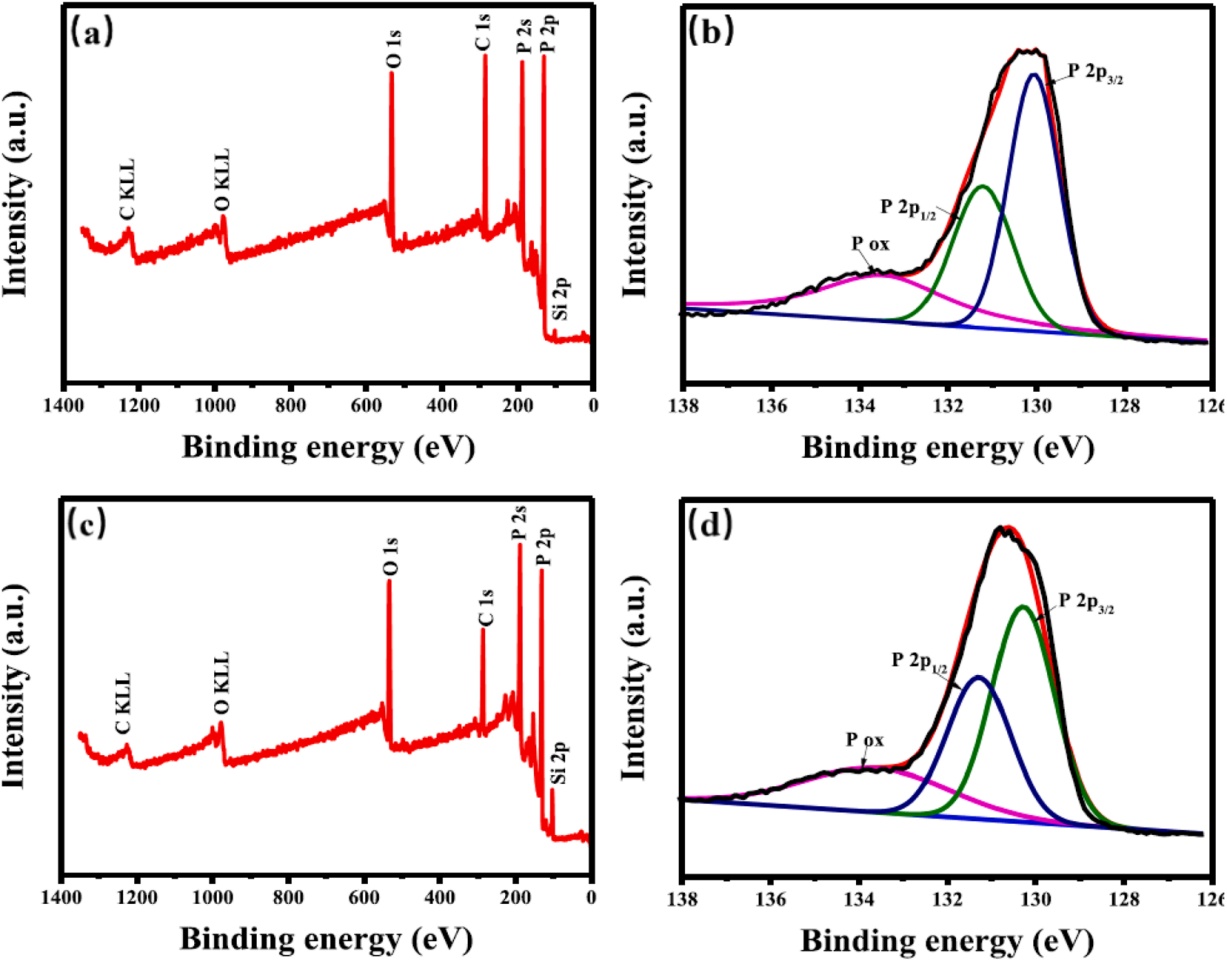
Survey spectra of holey phosphorene at (a) 4 V and (c) 3 V; high-resolution P 2p spectra at (b) 4 V and (d) 3 V. Reproduced from ref. 86 with permission from Wiley-VCH Verlag GmbH & Co. KGaA, Weinheim, *Angew. Chem., Int. Ed.*, 2017, 56, 10443–10445, © 2017 Wiley-VCH.^86^

Fig. 13 presents the survey spectra of holey phosphorene at (a) 4 V and (c) 3 V, along with the high-resolution P2p spectra at (b) 4 V and (d) 3 V. Holey phosphorene was analyzed using XPS spectra to assess its surface composition and oxidation state. The full spectrum reveals the presence of elemental phosphorus (Fig. 13a–c), suggesting that the material could be BP. The Si 2p peaks observed are likely from small quartz particles introduced during the BP preparation, as indicated by the XPS survey spectra. At 130.0 eV (2p_3/2_) and 131.2 eV (2p_1/2_), the material exhibits doublets characteristic of crystalline BP. This indicates that the low-temperature electrochemical exfoliation method effectively preserves the structural integrity of the material and effectively minimizes the oxidation of holey phosphorene, improving its overall quality.

### 4.5 Transmission electron microscopy (TEM) of phosphorene

**Fig. 14:**
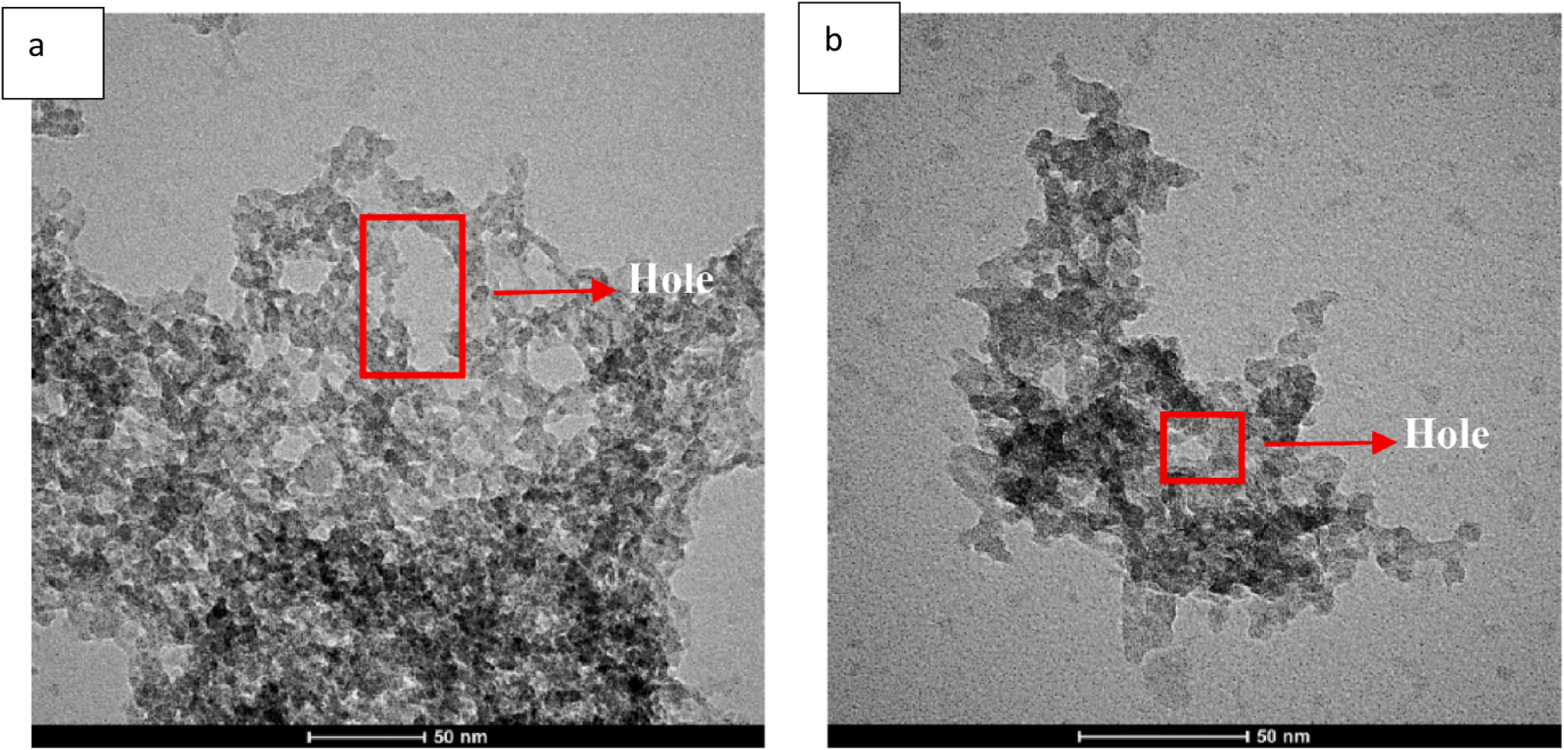
(a) Image of holey phosphorene obtained at 4 V and (b) image of holey phosphorene obtained at 3 V. Adapted from ref. 87, *Electrochemistry Communications*, 2021, 128, 107074. This Open Access Article is licensed under a Creative Commons Attribution-NonCommercial-No Derivatives 4.0 International (CC BY-NC-ND 4.0) Licence.^87^

The images in Fig. 14(a) (4 V) and Fig. 14(b) (3 V) show that holey phosphorene produced at 4 V has thinner sheets and larger holes (marked in red) compared to those made at 3 V as observed in TEM. The sample prepared at 4 V shows a smaller horizontal size and thickness than that prepared at 3 V, indicating differences in the holey phosphorene. This suggests that higher voltage increases the oxidation rate, reduces horizontal dimensions, and, along with larger pores and thinner sheets, characterizes the holey phosphorene resulting from the 4 V preparation.

## 5. Properties analysis of phosphorene

Analysis of phosphorene's properties reveals its unique characteristics, including a tunable bandgap that varies with layer thickness, making it well-suited for electronic and optoelectronic applications. It exhibits high carrier mobility, which is advantageous for fast electronic devices. Phosphorene exhibits anisotropic thermal and electrical conductivities, meaning its properties differ based on the direction in which they are measured. Despite these strengths, its environmental sensitivity, particularly to oxygen and moisture, poses challenges for stability. Advanced coatings and encapsulation techniques are being explored to enhance durability while preserving desirable properties.

### 5.1 Key properties of phosphorene

Table 1 summarizes the critical properties of phosphorene. This table provides the essential properties of phosphorene, which help understand its potential applications and limitations in various fields.

**Table 1:** Classification of the major properties exhibited by phosphorene. Adapted from ref. 88, *Nat. Commun.*, 2014, 5, 4475. This article is available under the Creative Commons Attribution-NonCommercial-NoDerivatives (CC BY-NC-ND) license and permits non-commercial use of the work^88^

Properties	Insights	Reference
Crystal structure	Orthorhombic	88
Layer thickness	∼0.85 nm per monolayer	
Bandgap	∼0.3 eV in its bulk form to approximately 1.5 eV	
Electrical conductivity	High anisotropy; conductance varies with crystal direction	
Carrier mobility	As high as 1000 to 10 000 cm^2^ V^−1^ s^−1^	
Mechanical properties	High flexibility; Young's modulus around 24–44 GPa	
Thermal conductivity	Anisotropic; ∼30 W m^−1^ K^−1^ (in-plane) and significantly lower out-of-plane	
Optical properties	Intense absorption in the visible to near-infrared (NIR) spectrum; tunable photoluminescence	
Stability	Degrades under ambient conditions; sensitive to oxygen and moisture	
Chemical reactivity	Reacts with oxygen and water; can be stabilized with encapsulation or passivation	

### 5.2 Evaluation of different phosphorene synthesis approaches

Table 2 summarizes the major synthesis approaches for phosphorene, each differing in output quality, scalability, and process complexity. Mechanical exfoliation yields high-quality monolayer sheets but suffers from low production efficiency. Liquid-phase exfoliation enables larger-scale synthesis, although the resulting flakes typically exhibit lower structural quality and thickness control. Chemical vapor deposition (CVD) allows precise and uniform growth of phosphorene layers; however, the process is technically challenging and cost-intensive. Overall, each method offers a unique balance between material quality, production scale, and practical feasibility, depending on the intended application.

**Table 2:** Overview of phosphorene synthesis techniques and corresponding advantages

Synthesis technique	Advantage	Parameters	Reference
Mechanical exfoliation	Producing high-quality, defect-free phosphorene monolayers	Uses sticky tapes like Scotch Magic tape or blue nitro tape	89
Chemical vapor deposition (CVD)	Large-area growth of phosphorene with precise thickness and uniformity	Deposition using PH_3_, H_2_, or Ar. Temperature: 500–1000 °C. Pressure control. Substrate choice influences nucleation. Precise control over thickness, orientation	90
Electrochemical synthesis	Scalable and cost-effective method for producing high-quality phosphorene	Higher voltage: accelerates reactions, enhances exfoliation, and risks degradation. Lower voltage: safer, slower, longer exfoliation	91
Liquid-phase exfoliation (LPE)	Enables large production of phosphorene with the flexibility to produce various nanosheet sizes	Solvent: NMP. Sonication for exfoliation. Room temperature. The concentration of black phosphorus in a solvent	92
Isopropanol–water cosolvents	Improve the stability of dispersion and the effectiveness of exfoliation for phosphorene	The presence of water affects stability	93
Chemical vapor transport (CVT)	Offers high-quality, large-area phosphorene growth with well-controlled crystal size	High temperature, vacuum conditions, sealed quartz tube	94

### 5.3 Differences between various phosphorenes

Table 3 shows the differences and similarities among black phosphorus, few-layer black phosphorous, red phosphorus, and phosphorene, particularly in their structural and electronic characteristics.

**Table 3:** Comparison among different allotropes and forms of phosphorus: black phosphorus, few-layer black phosphorus, red phosphorus, and phosphorene^95,96^

Properties	Black phosphorous	Few-layer black phosphorus	Red phosphorous	Phosphorene
Bandgap	∼0.3 eV (bulk) to ∼2 eV (monolayer)	∼0.3 eV (bulk) to ∼1.5 eV (few layers)	∼1.5 eV	∼0.3 eV (bulk) to ∼2 eV (monolayer)
Electrical conductivity	Semiconductor	Semiconductor with good conductivity	Insulator to semiconductor	Semiconductor
Thermal conductivity	Anisotropic	Anisotropic	Lower than black phosphorus	Anisotropic
Structure	Layered, puckered sheets	Layered structure	Amorphous or crystalline form	A single layer of black phosphorus
Carrier mobility	High: up to ∼1000 cm^2^ V^−1^ s^−1^	High: can reach up to ∼1000 cm^2^ V^−1^ s^−1^	Low compared to black phosphorus	High: comparable to black phosphorus, up to ∼1000 cm^2^ V^−1^ s^−1^
Thickness	It can be exfoliated to single or few layers	Few-layer (2–5 layers)	Typically, bulk material, not layered	Monolayer (single atomic layer)
Stability	Moderately stable in inert environments	Less stable than bulk BP; medium reactivity	More stable than white phosphorus	Less stable than bulk BP and few-layer BP

### 5.4 Applications of phosphorene

Phosphorene, a single layer of black phosphorus, has garnered significant attention due to its unique electronic, optical, and mechanical properties. This two-dimensional (2D) material shows great promise in a wide range of applications:

(1) Transistors and semiconductors: phosphorene's high carrier mobility (up to 1000 cm^2^ V^−1^ s^−1^) and tunable direct bandgap (ranging from ∼0.3 eV in the bulk to ∼2 eV in monolayers) make it ideal for field-effect transistors (FETs).^97^ These properties enable high-speed electronic devices and could surpass silicon in specific applications, particularly in flexible and wearable electronics and optoelectronics: the direct bandgap and broad-spectrum absorption from visible to infrared make phosphorene suitable for photodetectors and other optoelectronic devices. Its ability to efficiently convert light into electrical signals can be exploited in high-performance imaging systems and light sensors.^98^

(2) Solar cell phosphorene can be used in photovoltaic devices due to its high absorption coefficient and suitable bandgap.^99^ It can potentially enhance solar cells' efficiency, particularly in tandem solar cells, where it can complement other materials to capture a broader range of the solar spectrum.^100^

(3) Energy storage: with its high surface area and excellent electrical conductivity, phosphorene is promising for energy storage applications such as batteries and supercapacitors.^101^ Its ability to intercalate ions efficiently makes it a candidate for anodes in lithium-ion batteries, potentially offering higher capacity and faster charging times than conventional materials.^102^

(4) Thermoelectric devices: the thermal conductivity and high electrical conductivity of phosphorene make it suitable for thermoelectric applications, which can convert waste heat into electrical energy.^102^ This could lead to more efficient power generation systems and enhanced cooling technologies.

(5) Sensors: phosphorene's sensitivity to environmental changes makes it an excellent material for gas, biosensors, and chemical sensors.^103^ Its large surface area and high reactivity allow for detecting minute environmental changes, making it valuable in ecological monitoring and medical diagnostics.

(6) Biomedical applications: phosphorene's unique electronic properties, which are biocompatible, open up possibilities in the biomedical field, such as drug delivery systems and bioimaging. Phosphorene-based materials can be used for targeted drug delivery, leveraging their ability to interact with biological molecules and release drugs at specific sites within the body.^104^

## 6. Analysis of the phosphorene coating technique

Phosphorene, a single layer of black phosphorus, has remarkable electronic, optical, and mechanical properties, making it an attractive material for various applications, including coatings. However, phosphorene's stability and effective integration as a coating material pose significant challenges. Several techniques have been developed to coat and protect phosphorene, ensuring its stability and enhancing its performance in practical applications.

### 6.1 Encapsulation with polymer coatings

**Fig. 15:**
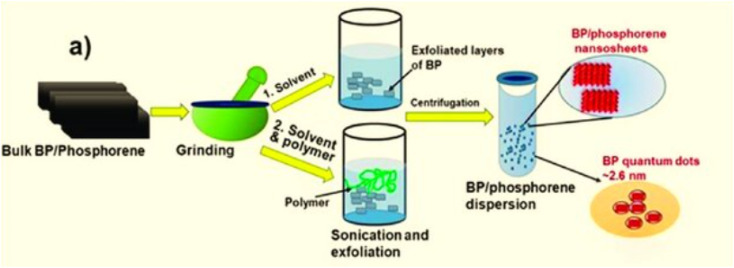
Diagram illustrating polymer encapsulation of phosphorene for passivation against ambient degradation. Reproduced from ref. 105 with permission from the American Chemical Society under License No. 6203700418319, *Nano Lett.*, 2014, 14(12), 6964–6970, © 2014 American Chemical Society.^105^

Fig. 15 illustrates the polymer encapsulation process of phosphorene. Encapsulation with polymers is one of the most effective techniques to protect phosphorene from degradation due to environmental exposure. Polymers such as poly(methyl methacrylate) (PMMA) and hexagonal boron nitride (h-BN) are commonly used, as shown in Fig. 15. These polymers form a protective layer over phosphorene,^105^ preventing oxidation and moisture absorption. The encapsulation process typically involves spin coating, where a polymer solution is spun at high speeds to create a uniform thin film over the phosphorene layer.

### 6.2 Atomic layer deposition (ALD)

**Fig. 16:**
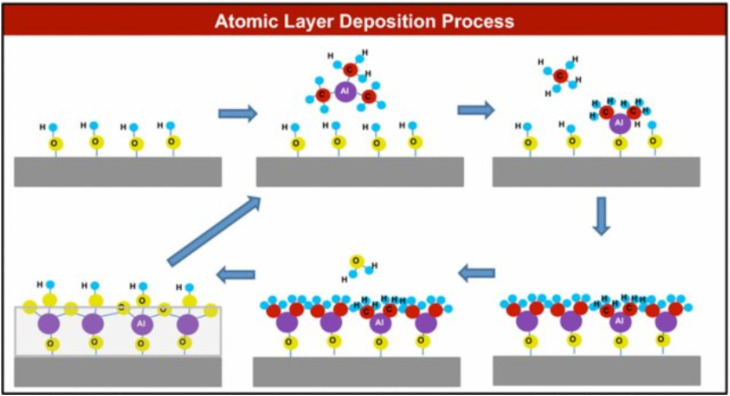
Schematic illustration of one cycle of Al_2_O_3_ atomic layer deposition (ALD) growth. Adapted from ref. 106, *Heritage Science*, 2015, 3, 1–12. This Open Access Article is distributed under the terms of the Creative Commons Attribution (CC BY) License.^106^

Atomic layer deposition (ALD) deposits thin films with atomic-level precision. ALD can coat phosphorene with materials such as aluminum oxide (Al_2_O_3_) or hafnium oxide (HfO_2_), creating a robust protective layer.^106^ This method ensures a conformal coating that encapsulates phosphorene, protecting it from environmental factors while maintaining its electronic properties. The ALD process in Fig. 16 involves alternating pulses of precursor gases that react with the surface, forming a thin, uniform film.

### 6.3 Chemical vapor deposition (CVD)

**Fig. 17:**
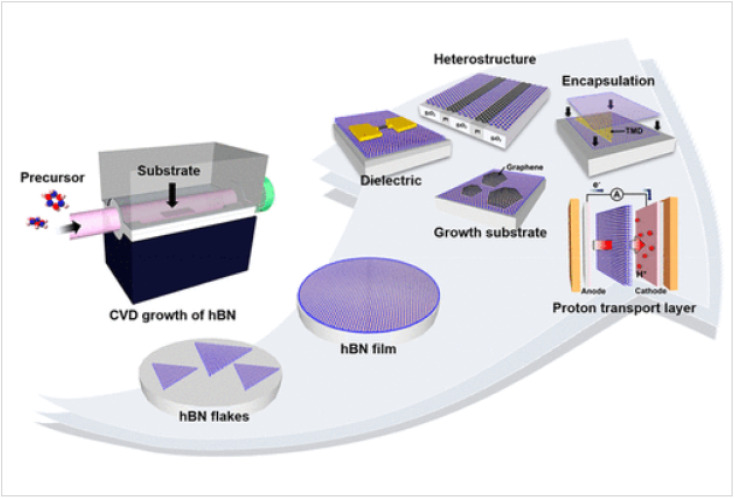
Schematic illustration of chemical vapor deposition (CVD) growth of hexagonal boron nitride (h-BN). Reproduced from ref. 107 with permission from the American Chemical Society, *Accounts of Materials Research*, 2022, 3(7), 748–760, © 2022 American Chemical Society.^107^

Chemical vapor deposition (CVD) can deposit protective coatings over phosphorene. Materials such as graphene and hexagonal boron nitride (h-BN) can be grown directly on phosphorene using CVD, as shown in Fig. 17. This method provides excellent coverage and adhesion, forming a protective barrier that enhances phosphorene's stability and electronic performance.^107^ CVD involves the chemical reaction of vapor-phase precursors on the substrate surface at high temperatures, resulting in the deposition of a thin film.

### 6.4 Sol–gel coating

**Fig. 18:**
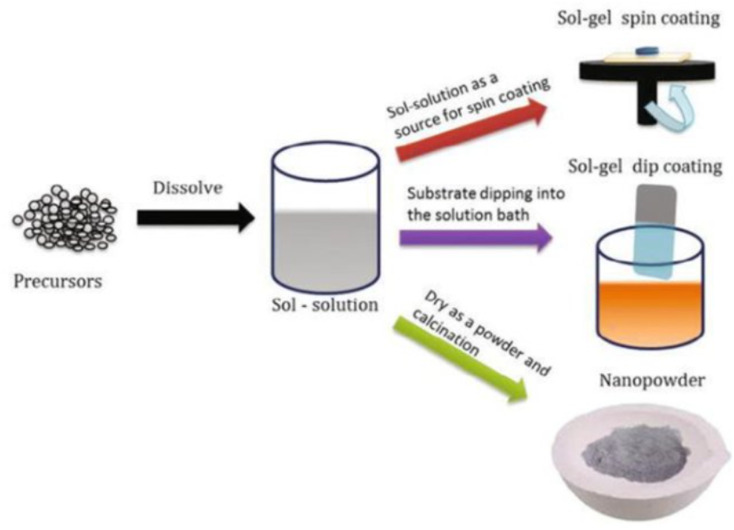
Schematic diagram illustrating the sol–gel coating process. Adapted from ref. 108, Thiagarajan *et al.*, *Recent Applications in Sol–Gel Synthesis*, 2017, pp. 1–17. This content is distributed under the terms of the Creative Commons Attribution 3.0 Unported Licence, which permits commercial use, distribution, and reproduction, provided the original authors and source are appropriately acknowledged.^108^

Sol–gel techniques involve the preparation of a colloidal solution (sol) that subsequently undergoes gelation to form a three-dimensional network (gel) containing the desired coating material.^108^ This method can be used to coat phosphorene with various oxide materials, providing protection and improving its mechanical properties, as shown in Fig. 18. The sol–gel process typically includes steps such as hydrolysis and condensation reactions, followed by drying and heat treatment to form a dense, uniform coating.

### 6.5 Layer-by-layer (LbL) assembly

**Fig. 19:**
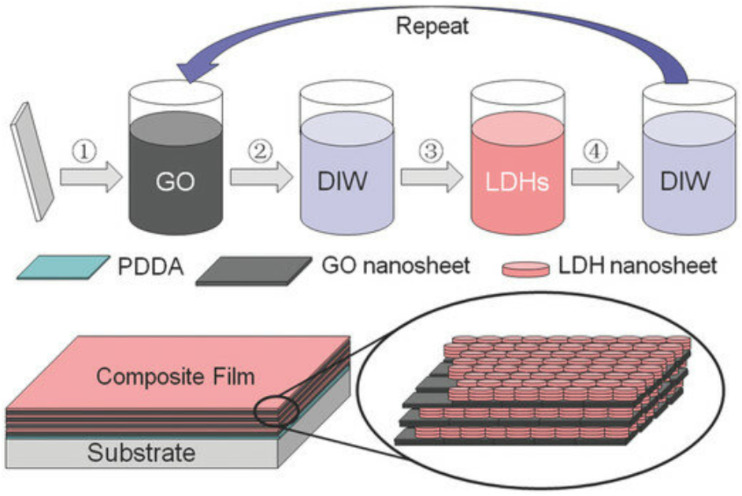
Schematic illustration of the layer-by-layer assembly process (top) and the resulting layered structure (bottom) of graphene oxide/layered double hydroxide (GO/LDH). Adapted from ref. 109, *Small*, 2016, 12, 745–755. This Open Access Article is distributed under the terms of the Creative Commons license.^109^

Layer-by-layer (LbL) assembly is a versatile technique for creating multilayer coatings by alternating the deposition of positively and negatively charged materials, as shown in Fig. 19. This method can be used to coat phosphorene with various polymers, nanoparticles, or other 2D materials. LbL assembly allows for precise control over the thickness and composition of the coating, enhancing the protective and functional properties of phosphorene.^109^ The process involves the sequential dipping or spraying of phosphorene into solutions of the coating materials, with washing steps in between to remove excess materials.

### 6.6 Electrophoretic deposition (EPD)

**Fig. 20:**
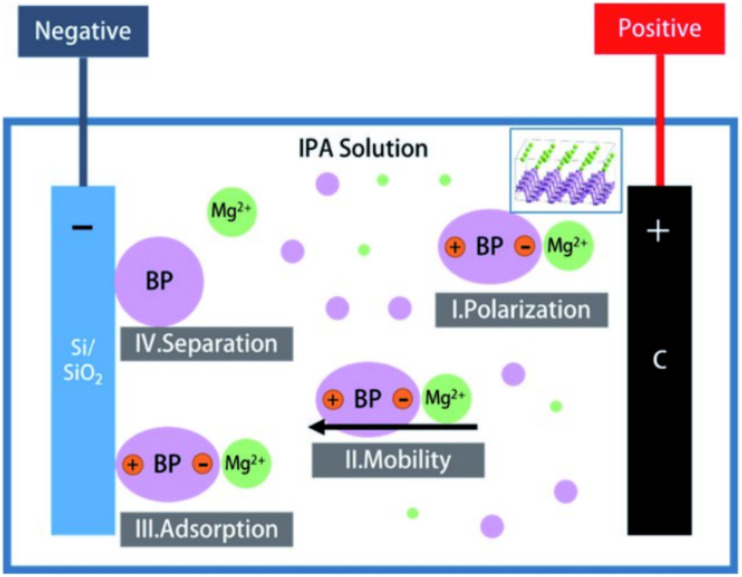
Schematic illustration of the mechanism for coating by electrophoretic deposition. Adapted from ref. 110, *RSC Adv.*, 2020, 10, 13379–13385. This Open Access Article is licensed under a Creative Commons Attribution 3.0 Unported Licence.^110^

Electrophoretic deposition (EPD) is a technique where charged particles suspended in a liquid medium are deposited onto a substrate under the influence of an electric field shown in Fig. 20. EPD can coat phosphorene with various nanoparticles or polymers, forming a uniform and adherent coating. This method is advantageous for its simplicity, scalability, and ability to coat complex shapes and surfaces.^110^ The EPD process involves applying a voltage between two electrodes submerged in the suspension, causing the charged particles to migrate and deposit onto the substrate.

## 7. Properties analysis of phosphorene coatings

Phosphorene, a monolayer form of black phosphorus, exhibits exceptional electronic and optical properties, but its sensitivity to environmental factors limits its practical applications. To address this, various coating techniques have been employed to enhance its stability and performance. This analysis focuses on the characterization of these coatings, examining how different techniques impact the properties of phosphorene.

### 7.1 Analysis of scanning electron microscopy (SEM)

**Fig. 21:**
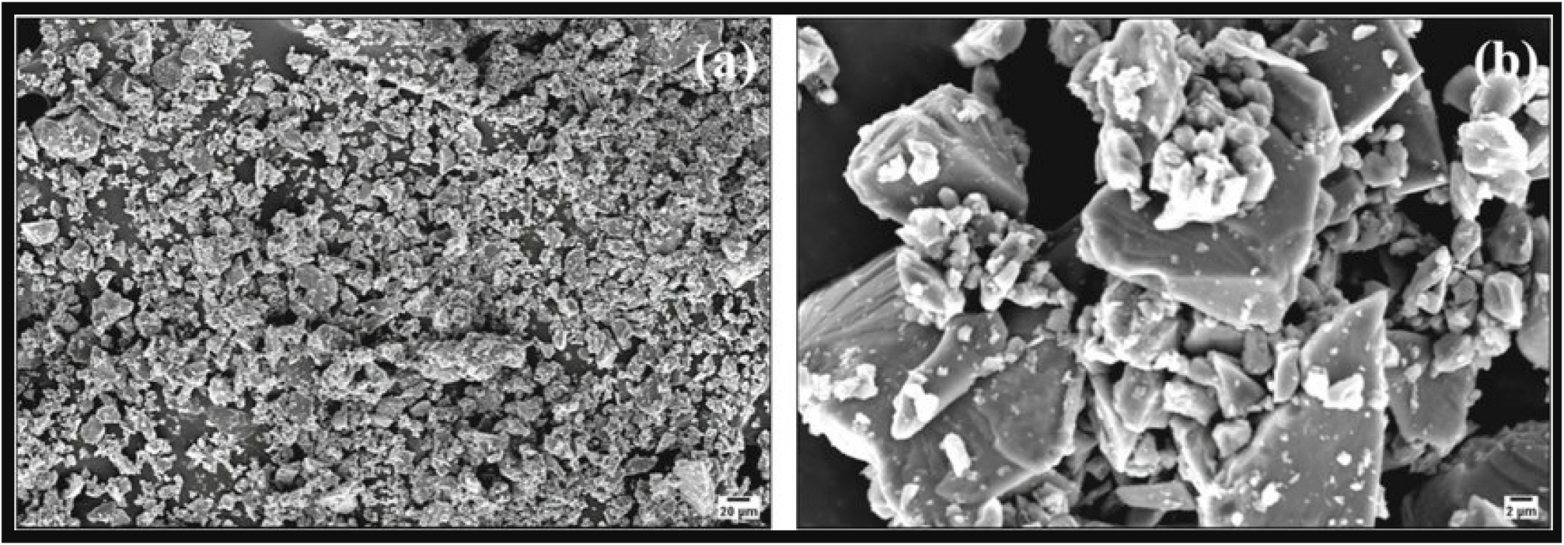
(a and b) SEM images of red phosphorus showing microsized particles with irregular morphologies. Adapted from ref. 111, *Small Methods*, 2017, 1, 1700260. This Open Access Article is distributed under the terms of the Creative Commons license.^111^

Fig. 21(a) displays a low-magnification SEM micrograph showing a highly fragmented and irregular morphology distributed over the substrate surface. The sample consists of numerous micron-scale particles with sharp edges and rough surfaces, indicating incomplete exfoliation of bulk phosphorus. The granular texture and non-uniform particle size distribution suggest that the mechanical or chemical processing steps primarily led to the formation of phosphorus micro-flakes and debris-like aggregates rather than well-defined layered structures. Such irregular fragmentation has been reported in the initial stages of black phosphorus exfoliation, where shear forces and partial chemical etching break the parent crystal into randomly shaped micro-domains.^111^

In contrast, Fig. 21(b) shows a higher-magnification view revealing more distinct layered platelet-like structures surrounded by smaller agglomerated particles. The larger platelets exhibit flat facets and angular edges characteristic of layered phosphorus crystallites, suggesting partial success in delamination. The presence of numerous small clusters attached to the platelet surfaces may originate from residual reaction by-products or re-agglomeration during washing and drying steps. Similar hierarchical structures—large phosphorus flakes decorated with nanoscale residues—have been observed in chemically exfoliated black phosphorus systems.

### 7.2 Analysis of atomic force microscopy (AFM)

**Fig. 22:**
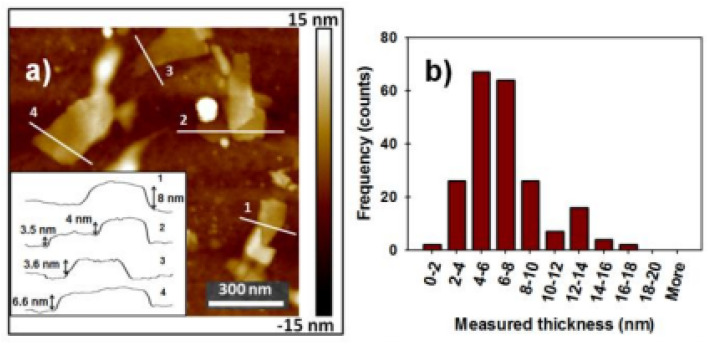
(a) AFM image of microwave-exfoliated few-layer black phosphorus (FL-BP); the inset shows the corresponding height profiles. (b) Histogram of thickness values obtained from more than 200 measurements. Adapted from ref. 112, *Nano Res.*, 2014, 7, 853. This figure is reused from an open-access publication.^112^

The AFM results (Fig. 22a and b) indicate that the measured heights of the FL-BP sheets range from approximately 2 nm to 15 nm, confirming the presence of few-layer to multilayer phosphorene (corresponding to ∼2–18 layers). Consistent with the NIR observations, the yield of bilayer phosphorene was relatively low compared to thicker few-layer sheets (4–6 layers). When bilayer regions were detected, they were typically located near the edges of larger, thicker flakes. From the height distribution shown in Fig. 22(b), the average thickness of the analyzed flakes was determined to be 6.5 ± 2.6 nm.^112^

### 7.3 Analysis of Raman spectroscopy

**Fig. 23:**
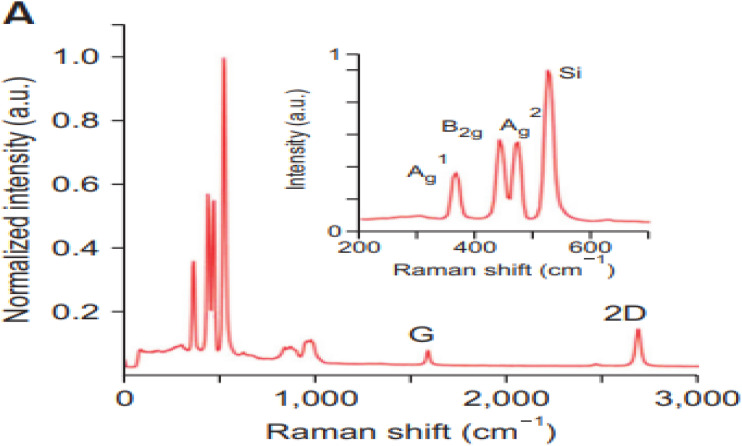
Raman spectrum of black phosphorus (BP) flakes showing the characteristic BP vibrational modes. Adapted from ref. 113, *Nat. Commun.*, 2015, 6, 6647. This figure is reused in an Open Access publication with appropriate acknowledgment.^113^

Fig. 23 presents the Raman spectrum of black phosphorus (BP) flakes, highlighting the characteristic BP peaks. Raman is used to analyze the vibrational modes of both phosphorene and its coating. This technique provides information on chemical composition, quality of the coating, and any strain or defects present. By comparing Raman spectra of coated phosphorene with those of uncoated samples, one can assess how the coating affects the electronic and optical properties of phosphorene. The Raman spectra of the passivation materials remain unchanged during the exposure period and are unaffected by the BP flakes,^113^ suggesting the presence of physical bonds (van der Waals interactions) between the passivating layers and the BP flake (Fig. 23). As a result, the 2D materials (graphene and hBN) effectively prevent oxygen and water from penetrating and degrading the BP flakes.

### 7.4 Analysis of X-ray photoelectron spectroscopy (XPS)

**Fig. 24:**
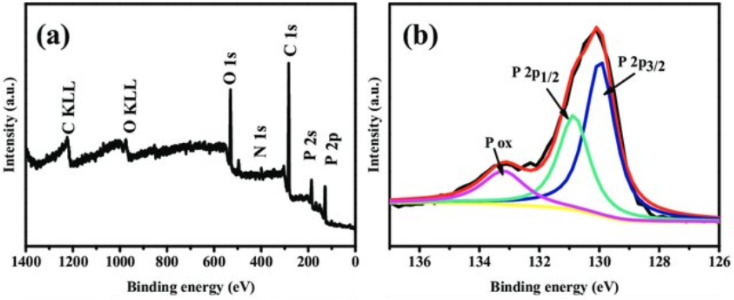
(a) XPS survey spectra of holey phosphorene and (b) high-resolution XPS spectra of the P 2p core level for holey phosphorene. Adapted from ref. 114, *FlatChem*, 2017, 2, 49–53. This figure is reused in an Open Access publication with appropriate acknowledgment.^114^

XPS is employed to investigate the coating material's chemical composition and electronic state. XPS helps identify elemental composition, oxidation states, and chemical bonding environments, which are critical for understanding how the coating interacts with phosphorene. Fig. 24 displays (a) the XPS survey spectra of holey phosphorene and (b) the high-resolution XPS spectra of P2p for the holey phosphorene. For instance, XPS spectra can reveal any chemical changes or reactions occurring at the phosphorene–coating interface. The survey XPS spectra (Fig. 24a) showed no residual sodium in the holey material, aligning with the findings from the EDS spectrum. The high-resolution XPS spectra (Fig. 24b) displayed the P 2p_1/2_ and P 2p_3/2_ doublet at 130.9 eV and 129.9 eV, respectively, which are characteristic peaks of crystalline BP.^114^

### 7.5 Analysis of transmission electron microscopy (TEM)

**Fig. 25:**
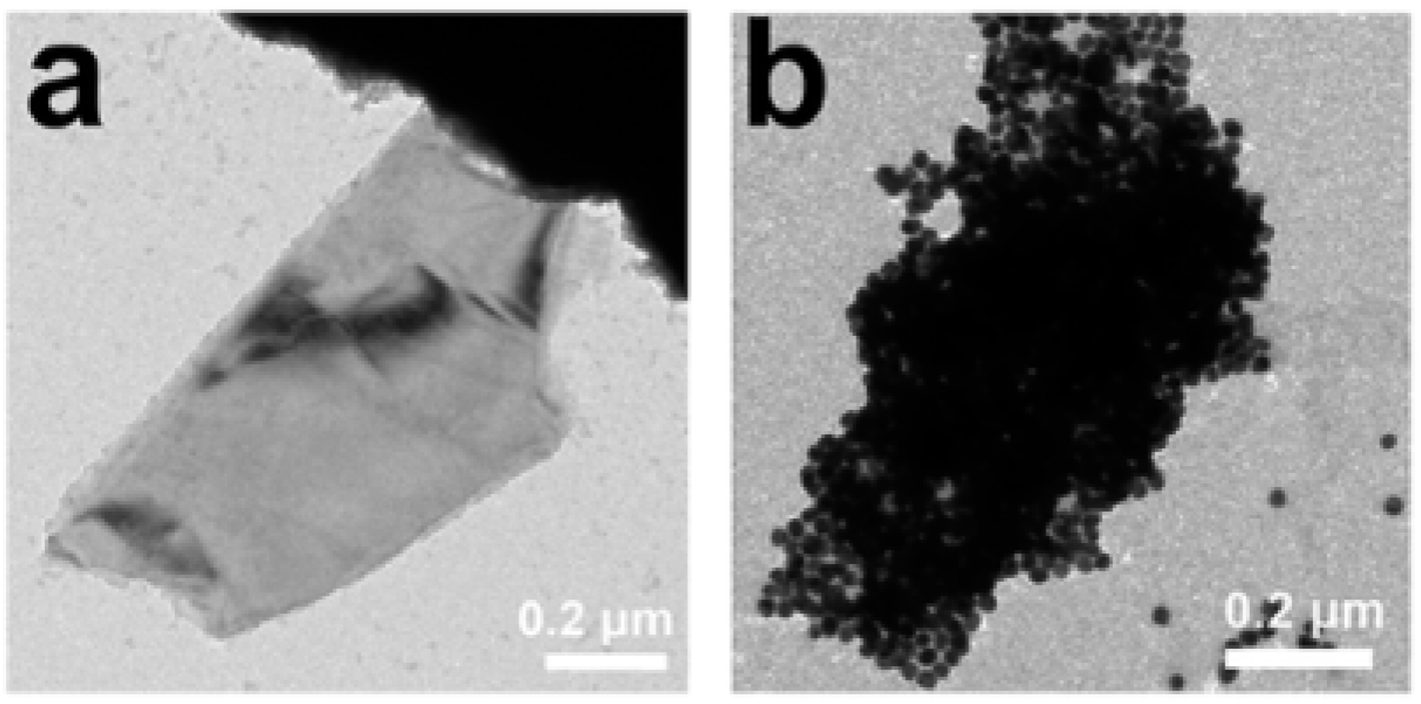
Transmission electron microscopy (TEM) images of black phosphorus: (a) BP sheet and (b) BP–AuNSs. Reproduced from ref. 115 with permission from the American Physical Society under License No. RNP/26/FEB/101982, *Phys. Rev. Lett.*, 2015, 114, 046801, © 2015 American Physical Society.^115^

Fig. 25(a) shows a thin, sheet-like structure characteristic of few-layer phosphorene. The flake appears semi-transparent under the electron beam, indicating a reduced number of layers, which is consistent with exfoliated black phosphorus nanosheets. The smooth surface morphology and uniform contrast suggest minimal structural defects or fragmentation within the observed region.^115^

In contrast, Fig. 25(b) displays a dense aggregation of nanosized particles, which likely indicates the formation of phosphorus-derived nanoparticles or degradation products (*e.g.*, P_*x*_O_*y*_ species). The particles appear to be well below 100 nm, forming clusters with high contrast due to their increased thickness or crystallinity. The stark difference between Fig. 25(a) and (b) suggests that the synthesis or post-treatment conditions significantly influence the final morphology—ranging from layered nanosheets to aggregated nanoparticles.

## 8. The research gap of earlier investigations of phosphorene

Phosphorene, a two-dimensional material derived from black phosphorus, has garnered significant interest due to its tunable bandgap and promising electronic properties. Despite the progress made, several research gaps in the synthesis and characterization of phosphorene are still present, particularly concerning the precise control and understanding of its bandgap formation.

One major challenge is the development of scalable and controlled synthesis methods. While mechanical exfoliation, liquid-phase exfoliation, and chemical vapor deposition (CVD) have been employed to synthesize phosphorene, each technique has limitations. Although mechanical exfoliation yields high-quality phosphorene, it lacks scalability for large-scale or industrial applications. Liquid-phase exfoliation offers better scalability but often results in lower quality with defects and inconsistent thickness control. Phosphorene is still in the early stages, facing issues related to substrate compatibility and the uniformity of the produced layers.

Another significant research gap is the stability and degradation of phosphorene. Phosphorene is known for its instability under ambient conditions, leading to rapid oxidation and degradation. Studies have shown that without proper encapsulation, phosphorene degrades quickly, which adversely affects its electronic properties and bandgap. More robust and practical techniques are needed to protect phosphorene while maintaining its desirable properties.

Achieving precise control over the bandgap of phosphorene remains a challenge. The tunable bandgap is one of its most attractive features, but thickness variations and defects during synthesis can lead to inconsistencies in the bandgap. Further research is required on the effects of strain, doping, and external fields on the bandgap of phosphorene. Theoretical studies must be complemented with experimental verification to develop reliable methods for bandgap tuning.

Characterization techniques for understanding bandgap formation and electronic properties of phosphorene also need refinement. Traditional methods such as Raman spectroscopy, atomic force microscopy (AFM), and transmission electron microscopy (TEM) have limitations in fully capturing the dynamic behavior and real-time changes in phosphorene under different conditions. There is a need to develop non-destructive methods to study the intrinsic properties of phosphorene more effectively. Scalable synthesis methods, improved stability, precise bandgap control, refined characterization techniques, and a deeper understanding of interlayer interactions are necessary to fully realize the potential of phosphorene in electronic and optoelectronic applications.

## 9. Future prospects and challenges of phosphorene

Future research on phosphorene should focus on developing scalable synthesis techniques, such as chemical vapor deposition (CVD), to produce a high-quality material consistently. Precise control over layer thickness is crucial, as the bandgap of phosphorene varies with the number of layers, making atomic layer deposition (ALD) a promising method to explore. Enhancing the stability of phosphorene is essential, as it is highly sensitive to environmental factors like oxygen and moisture; effective passivation and encapsulation strategies are necessary to preserve its properties. Advanced characterization methods, including *in situ* Raman spectroscopy and high-resolution transmission electron microscopy (HRTEM), should be employed to monitor the material during synthesis and device operation. Bandgap engineering through doping and strain application offers a promising avenue for tuning phosphorene's electronic properties to suit specific applications. Additionally, research should focus on integrating phosphorene into existing electronic and optoelectronic devices, ensuring compatibility with current fabrication processes. Exploring new coating materials and techniques can also help mitigate degradation, improving the long-term viability of phosphorene-based devices. Understanding and optimizing the interface between phosphorene and other materials in the device architecture will enhance performance. Finally, sustainable and environmentally friendly synthesis methods should be prioritized to minimize the ecological impact of large-scale phosphorene production. These efforts will be key to unlocking the full potential of phosphorene in advanced technological applications.

## 10. Conclusions

With its unique electronic and optical properties, phosphorene has emerged as a promising material for next-generation electronics and optoelectronics. Its tunable bandgap, high carrier mobility, and anisotropic properties offer significant advantages for applications ranging from transistors to photodetectors. However, the material's sensitivity to environmental factors like oxygen and moisture presents challenges that must be addressed for practical use. Ongoing research into scalable synthesis methods and precise layer control is essential for producing high-quality phosphorene on a larger scale. Stability enhancement through advanced coating and encapsulation techniques remains a critical area of focus for preserving phosphorene's properties over time.

Further exploration of bandgap engineering through doping and strain is necessary to tailor phosphorene for specific applications. Integrating phosphorene into the existing device architecture while ensuring compatibility with current fabrication processes is vital for commercial viability. Phosphorene, a two-dimensional material with remarkable electrical, thermal, and optical properties, is drawing attention for applications in electronics, energy storage, and biomedical devices. However, its development faces several significant challenges. One primary issue is its instability under ambient conditions; phosphorene readily degrades upon exposure to oxygen and moisture, which leads to rapid oxidation and loss in performance. This sensitivity necessitates a controlled environment during production, which complicates the development process. Another challenge lies in the scalability of phosphorene production. Current synthesis techniques, such as liquid-phase exfoliation and chemical vapor deposition (CVD), face limitations in achieving high-quality, uniform layers on a large scale, hindering the material's integration into industrial applications. Additionally, the environmental and health impacts of phosphorene production and disposal remain largely unknown. Potential toxicity from degradation products poses a concern, especially in biomedical applications where safety is paramount. Finally, integrating phosphorene with existing technologies requires extensive research, as well as the development of effective interfacial bonding techniques, to ensure stable and efficient device performance.

To address these challenges, several techniques show promise. One effective approach is surface passivation and encapsulation. By passivating the phosphorene surface with molecules or encapsulating it with stable materials like hexagonal boron nitride (hBN) or graphene, stability under ambient conditions can be significantly enhanced, thereby protecting phosphorene without compromising its electrical properties. Another promising technique is liquid-phase exfoliation with surfactants. This modified method not only helps achieve scalable production but also mitigates oxidation by employing surfactants that shield phosphorene from direct exposure to oxygen, slowing its degradation. These approaches, whether used individually or in combination, may offer the most viable solutions for addressing phosphorene's limitations, supporting its potential for future technological advancements.

## Conflicts of interest

The authors declared there is no conflict of interest.

## Data Availability

No primary research results, software or code have been included, and no new data were generated or analyzed as part of this review. All information cited within this review is derived from existing literature and publicly available sources.
